# Image quality in diagnostic radiology: a guide to methodologies for radiologists

**DOI:** 10.1590/0100-3984.2024.0088-en

**Published:** 2025-04-11

**Authors:** Andréa de Lima Bastos, Maria do Socorro Nogueira

**Affiliations:** 1 School of Medicine, Universidade Federal de Minas Gerais (UFMG), Belo Horizonte, MG, Brazil; 2 Comissão Nacional de Energia Nuclear (CNEN)/Centro de Desenvolvimento da Tecnologia Nuclear (CDTN), Seção de Dosimetria das Radiações (SECDOS), Belo Horizonte, MG, Brazil

**Keywords:** Quality control, Image processing, computer-assisted, Radiology, Diagnostic imaging, Practice guideline, Controle de qualidade, Processamento de imagem assistido por computador, Radiologia, Diagnóstico por imagem, Guia de prática clínica

## Abstract

The aim of this article is to provide a comprehensive guide to image quality assessment in diagnostic radiology, emphasizing practical methodologies for radiologists. The goal is to improve diagnostic accuracy and patient care on the basis of the understanding and application of quantitative and qualitative metrics in clinical practice and research. We conducted a review of the literature in the PubMed, Scopus, Web of Science, and Embase databases. The search terms included “image quality in radiology”, “quantitative and qualitative assessment”, “modulation transfer function”, “signal-to-noise ratio”, “contrast-to-noise ratio”, “radiation dose optimization”, and “artificial intelligence in image quality assessment”. The review identified the main methodologies for image quality assessment. We analyzed these metrics for their applicability in clinical settings, highlighting their benefits and limitations. In addition, we discuss qualitative methods such as visual assessment, the assessment of contrast/density, and peer review. This guide fills a gap in the literature by providing accessible, practical knowledge for general radiologists. Ongoing research, education, and technological development are essential to advance the field and ensure high standards in radiology practice.

## Abstract (pt)

Este artigo tem como objetivo fornecer um guia abrangente para a avaliação da qualidade da imagem na radiologia diagnóstica, enfatizando metodologias práticas para os radiologistas. A finalidade é aprimorar a precisão diagnóstica e o cuidado com o paciente com base no entendimento e aplicação de métricas quantitativas e qualitativas na prática clínica e na pesquisa. Foi realizada uma revisão utilizando as bases de dados PubMed, Scopus, Web of Science e Embase. A pesquisa incluiu termos como “qualidade da imagem na radiologia”, “avaliação quantitativa e qualitativa”, “função de transferência de modulação”, “relação sinal-ruído”, “relação contraste-ruído”, “otimização da dose de radiação” e “inteligência artificial na avaliação da qualidade da imagem”. A revisão identificou as principais metodologias para a avaliação da qualidade da imagem. Essas métricas foram analisadas quanto à sua aplicabilidade em ambientes clínicos, destacando seus benefícios e limitações. Além disso, foram discutidos métodos qualitativos, como avaliação visual, avaliação de contraste e densidade e avaliação por pares. Este guia preenche uma lacuna na literatura ao fornecer conhecimento acessível e prático para radiologistas em geral. A pesquisa contínua, a educação e o desenvolvimento tecnológico são essenciais para o avanço do campo e para garantir altos padrões de prática radiológica.

## INTRODUCTION

The continuous evolution of diagnostic radiology demands a deep understanding of image quality, especially in technologies that employ ionizing radiation, to ensure accurate and safe diagnostic processes^(1)^. Elements such as the technology used, the expertise of the radiologist, and the clinical condition of the patient play a role in determining the quality of the images obtained. In addition, digital transformation, advances in computing, and the integration of artificial intelligence into radiology practice have made it imperative to understand the parameters that influence image quality^(2,3)^. Therefore, radiologists must evaluate, recognize, and attempt to improve image quality, in clinical practice and in research. It is not enough for this competence to be limited to “reading images”; it is necessary to understand the technology used, to be familiar with the image quality control criteria, and to balance image quality with patient safety, which is a priority when performing diagnostic procedures, according to the “as low as reasonably achievable” principles^(4)^.

Image quality analysis encompasses not only quantitative metrics, which provide objective information, but also qualitative assessments, which are based on knowledge acquired through experience and continuing education^(2,5)^.

Metrics for assessing image quality should be aligned with the radiologist’s perception of an ideal image. This alignment ensures that metrics facilitate the differentiation between health and disease, the identification of diagnostically relevant structures and their characteristics, the classification of various abnormalities, and the reliable detection of relevant structures in the images^(4,6)^.

Despite the importance of the topic, there is a lack of studies in the literature that combine quantitative and qualitative methods for assessing image quality in a manner that is accessible to radiologists. This gap presents a challenge for professional practice, highlighting the need for a comprehensive guide.

Given the need for a practical guide to methodologies for image quality analysis, the aim of this article is to provide an overview of the main methodologies for assessing image quality in diagnostic radiology. The principles and applications of the most common metrics will be explored, and their applicability in medical practice and research will be discussed, as will how they can improve patient care standards and diagnostic accuracy. Our goal is to provide general radiologists with practical knowledge to assess image quality, interpret the results, and apply them in clinical practice and research proposals, thus promoting the ongoing improvement of diagnostic radiology. The main methodologies for image quality evaluation described in this article are presented in Table 1.

**Table 1:** Main methodologies for image quality assessment.

Image quality assessment methodologies	Main features
Quantitative Modulation transfer function	Measures the ability of the imaging system to reproduce contrast details^(7)^
Signal-to-noise ratio	Compares the useful signal level with the variation in background noise, higher values indicating better-defined images^(8)^
Contrast-to-noise ratio	Quantifies the difference between the signal of interest and background noise^(8)^
Detective quantum efficiency	Measures the efficiency of the imaging system in converting radiation into a useful image, taking into account image quality and radiation dose^(8)^
Image uniformity Qualitative Visual assessment	Measures the consistency of the imaging system response across the entire region under study^(9)^ Subjective analysis performed by experienced radiologists, focusing on factors such as noise, artifacts, sharp-ness, and overall image clarity^(10)^
Contrast and density Anatomical detail	Evaluates the contrast and density of anatomical structures in the images^(11)^ Assesses the visibility of anatomical structures, lesions, and a noma lies^(11)^
Peer review	Specialized professionals analyze the images and give their impressions on the image quality^(12)^

## METHODS

This review followed the Preferred Reporting Items for Systematic Reviews and Meta-Analyses guidelines, using the strategy as methodological guidance, given that the objective was to conduct a narrative review. There was no previous registration of this review on systematic review registration platforms.

Two reviewers, working independently, selected relevant works by searching the PubMed, Scopus, Web of Science, and Embase databases. The search terms used were designed to cover a broad spectrum of the literature related to image quality in radiology, including “image quality in radiology”, “quantitative and qualitative assessment of image quality”, “modulation transfer function” (MTF), “signal-to-noise ratio” (SNR), “contrast-to-noise ratio” (CNR), “radiation dose optimization in radiology”, and “artificial intelligence in image quality assessment”.

We included only works published in English, including original articles, review articles, and book chapters, as well as official guidelines issued by boards and committees that guide radiology practice. Conference abstracts were excluded, as were letters to the editor without original data and studies that did not specifically address the assessment of image quality in diagnostic imaging using ionizing radiation.

The collected evidence was narratively synthesized to compile the existing knowledge on methodologies for image quality assessment in diagnostic radiology. Quantitative and qualitative techniques, as well as their applications, benefits, and limitations, were highlighted to provide a detailed overview of these assessment methodologies, emphasizing the main techniques and strategies adopted in clinical practice and in research.

## RESULTS

**Figure 1:**
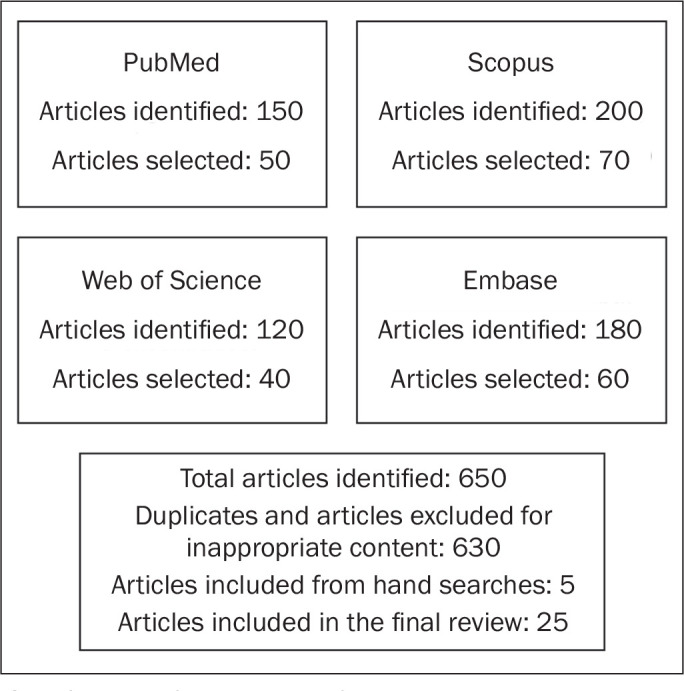
Process of selecting articles for review.

The search strategy for this article sought to identify relevant works on methodologies for image quality assessment in diagnostic radiology. The results are presented in Figure 1, which provides a visual representation of the process of selecting articles for the study and shows the initial number of articles obtained per database, the number of duplicates, the number of articles with inappropriate content or limited relevance to the scope of the review that were removed, the number of articles included by hand search, and the final number of articles included.

### Physical foundations of imaging in diagnostic radiology

Diagnostic radiology is based on fundamental physical principles for the generation, detection, and interpretation of medical imaging findings. Understanding the physical factors that affect image quality is crucial for the development of diagnostic imaging methodologies and for their efficiency in the clinical context, to reduce patient exposure to radiation. The quality of medical images depends on the following basic factors: image contrast, spatial resolution, image noise, and artifacts^(13)^. In general terms, these principles can be summarized as follows.

**Interaction of radiation with matter** - The basis of X-ray imaging techniques is the interaction between radiation and matter. This interaction can result in photoelectric absorption, the Compton effect, or pair production, varying according to the radiation energy and the type of tissue. The anatomical representation of the patient is made possible by the differential attenuation of radiation by various tissues^(14,15)^.

**Formation of a radiographic image** - A radiographic image is created by the spatial distribution of photons that pass through the object and reach the detector. The quality of such images is therefore influenced by factors resulting from that distribution, such as noise, spatial resolution, and contrast^(14,15)^.

**Contrast and density** - Image contrast, characterized by the difference in radiographic density between different regions, is influenced by tissue attenuation coefficients and the radiographic technique used. Density, which indicates the opacity or transparency of an area, reflects the amount of radiation absorbed by the tissue and that reaching the detector. Radiographic techniques can modify contrast and density, affecting the visualization of details^(13-15)^.

**Spatial resolution** - The spatial resolution, which relates to the ability to distinguish fine details, is influenced by the focus of the X-ray tube, the object-detector distance, the source-object distance, and detector characteristics such as pixel size^(13,15)^.

**Image noise** - Noise, which represents unwanted variations in the image, can impede interpretation and diagnosis^(13)^. Its most common types include:

• *quantum noise* (photon noise) - statistical variation in the number of photons reaching the detector, more noticeable at low radiation doses

• *electronic noise* - associated with the quality of the detection and image processing equipment

• *structural noise* (texture noise) - variations in the texture of the tissue itself that can be confused with pathologies

• *scattering noise* - reduces image contrast due to the scattering of photons in the object, where the scattered photons reach the detector without adding useful information about the structure of interest.

**Artifacts** - Artifacts can be defined as any structure seen in an image but which does not represent the actual anatomy^(4,13)^.

### Quantitative methodologies for image quality analysis

Quantitative analysis of the quality of diagnostic images is essential to ensure diagnostic accuracy and increase patient safety, using methods that provide measurable, objective data^(16)^. Below, we discuss the main quantitative techniques in this context.

#### MTF

*Definition* - The MTF measures the ability of the imaging system to reproduce details of contrast at various spatial frequencies, quantifying fidelity in the transmission of information from the object to the image^(7)^.

*Utility* - The MTF evaluates spatial resolution in imaging systems such as computed tomography (CT) and digital radiography^(7)^.

*Importance* - Understanding the MTF is crucial for adjusting equipment to maximize image quality and balance resolution and noise, which is essential for detecting pathologies^(7)^.

#### SNR

*Definition* - The SNR compares the useful signal level with the variation in background noise (variations that do not represent the image), higher values indicating images that are more well-defined^(7,8)^.

*Utility* - The SNR determines the quality of the image in terms of clarity and the ability to visualize fine details or lesions^(8)^.

*Importance* - Maintaining an adequate SNR is crucial for the visibility of subtle details without the need to increase the radiation dose^(8^.

#### CNR

*Definition* - The CNR quantifies the distinction between a signal of interest and background noise^(8)^.

*Utility* - The CNR assesses the ability of the image to differentiate structures with subtle contrast, i.e., where the contrast between the lesion and adjacent normal tissue is low.

*Importance* - In clinical practice, optimization of the CNR is essential to maximize image quality, allowing accurate visualization of anatomical and pathological details with minimal noise^(8)^.

#### Detective quantum efficiency

*Definition* - The detective quantum efficiency (DQE) measures the efficiency of an imaging system in converting radiation into a useful image, considering image quality and radiation dose^(8)^.

*Utility* - A high DQE value indicates that the system can produce high-quality images with a lower radiation dose.

*Importance* - The DQE facilitates the choice of equipment and settings that offer high-quality images with less radiation exposure, thus promoting patient safety^(8)^.

#### Image uniformity

*Definition* - Image uniformity is a measure of consistency in the response of the imaging system throughout the area under study, indicating the absence of unwanted variations that do not correspond to the actual scanned tissue and that could mimic or mask pathologies^(9)^.

*Utility* - Image uniformity is fundamental in modalities such as CT, in which signal variations can impact the diagnosis^(9)^.

*Importance* - High uniformity ensures that anatomical details and abnormalities are correctly visualized throughout the image, thus increasing diagnostic accuracy^(9)^.

### Applications of quantitative methodologies in radiology research

The use of quantitative methodologies in radiology research is essential to deepen the understanding and analysis of image quality, as well as to improve it, in addition to facilitating optimization of the radiation dose and increasing patient safety. Quantitative metrics offer an objective way to analyze data for scientific study proposals, with meaningful results. The adoption of these methodologies in radiology research can improve the quality of diagnoses, as well as increasing patient safety and the effectiveness of radiological procedures^(6)^.

Below, we discuss some applications of these methodologies in various research scenarios.

**Equipment performance assessment** - Quantitative methodologies are essential for analyzing the performance of new diagnostic imaging devices. Measurements of parameters such as the MTF, SNR, and DQE help determine whether the equipment can produce high-quality images with the lowest possible radiation dose. This assessment is essential to ensure that new technologies meet safety and efficacy criteria before they can be marketed^(8,17)^.

**Optimization of imaging protocols** - The application of quantitative methodologies allows researchers to optimize the imaging protocols for various diagnostic techniques. Quantitative analysis facilitates the identification of configurations that balance image quality and reduce radiation exposure, promoting safer and more effective radiology practice^(18,19)^.

**Development of image processing algorithms** - During the development of image processing algorithms, quantitative methodologies evaluate their efficiency in improving image quality. Indices such as the SNR and CNR are employed to quantify improvements in processed images, identifying the most effective processing techniques^(20)^.

**Radiation safety research** - In studies focused on radiation safety, quantitative methodologies are essential to explore the relationship between radiation dose and image quality. Such research helps to define safe radiation limits, encouraging practices that protect patients and health care professionals from unnecessary exposure^(21,22)^.

### Qualitative methodologies for image quality analysis

Qualitative metrics complement quantitative assessments by providing expert-level technical understanding, which is crucial for accurate radiological interpretation and for ensuring patient safety. These methodologies are essential for personalizing care by allowing imaging procedures to be adapted to specific needs, for ensuring patient safety, for training healthcare professionals to develop a keen sense of image assessment, for advancing technological development, and for promoting ongoing improvements in image quality. Some qualitative techniques and parameters are discussed below.

**Visual assessment** - Subjective visual assessment, which is essential in clinical practice, is performed by radiologists with experience in the relevant field. This assessment involves the analysis of factors such as noise, artifacts, sharpness, and overall image clarity^(10,23)^.

**Contrast and density** - It is important to assess the contrast and density of anatomical structures in the images. Adequate contrast and appropriate density levels are essential for diagnostic accuracy^(11)^.

**Anatomic detail** - An evaluation of the anatomical details assesses the visibility of anatomic structures, lesions, and abnormalities. The ability to discern fine details is crucial to the diagnosis^(11)^.

**Peer review** - Expert professionals review the images and provide their impressions of the image quality^(12,24)^.

### Applications of qualitative methodologies in radiology research

Qualitative methodologies provide important information about image quality in radiology, contributing to the improvement of imaging protocols and acquisition systems, as well as, consequently, to the provision of patient care. Some applications are highlighted below.

**Visual assessment** - Researchers can perform visual assessments to identify artifacts, evaluate noise, and determine image sharpness. This facilitates understanding of human perception of image quality and helps identify areas for improvement^(6,10)^.

**Identification and classification of artifacts** - Qualitative analysis allows the identification and classification of artifacts present in radiological images, such as motion artifacts, beam hardening, and metal artifacts. This facilitates understanding of the sources of image degradation^(6)^.

**Peer review** - Interviews, expert panels, and questionnaires can be employed to collect radiologist impressions of image quality. Their perceptions and opinions can provide valuable information for improving imaging protocols and acquisition systems^(6,25)^.

**Comparison with reference standards** - Qualitative methodologies can be employed to compare radiological images with established reference standards, such as image quality guidelines. This helps determine whether the images meet the quality standards required for diagnosis^(19,24)^.

## DISCUSSION

The results of this study initially provide a comprehensive overview of the fundamental physical principles that influence image quality in diagnostic radiology. Understanding the factors that affect image quality, such as contrast, spatial resolution, noise, and artifacts, is essential for the development of effective diagnostic imaging methodologies, the objective being to minimize patient exposure to radiation.

The interaction between radiation and matter is at the core of most diagnostic imaging techniques. The quality of the resulting image is a product of several factors, such as spatial resolution and noise levels, influenced not only by the imaging technique used but also by the characteristics of the equipment. The findings of the present study highlight the importance of understanding the attenuation of radiation by different tissues, as essential to generate high-quality anatomical representations^(13,14)^.

The contrast between tissues and the density of structures are essential to differentiate normal anatomy from that modified by pathologies, being influenced by attenuation coefficients and radiographic techniques. Inadequate contrast can result in missed diagnoses, especially in pathologies that still present discretely. Spatial resolution is essential to visualize fine anatomical details, whereas noise, especially quantum noise, can compromise image quality. Reducing such noise is crucial in low-dose radiation techniques^(13,15)^.

Quantitative methods are essential for assessing image quality, offering objective metrics such as the MTF, SNR, CNR, and DQE. High MTF values indicate better preservation of details, important for detecting small lesions, whereas a high SNR improves image definition and reduces background noise^(7,8)^. The CNR helps distinguish subtle contrasts between tissues, being crucial in modalities such as magnetic resonance imaging and CT. A high DQE is related to the production of high-quality images with lower radiation doses, which is especially relevant in sensitive patients, such as children^(7-9)^.

The adoption of quantitative methodologies for image quality analysis in research has profound implications. As demonstrated in the present study, quantitative techniques allow rigorous evaluation of imaging equipment and the development of optimized imaging protocols^(7)^. These metrics can also be used to evaluate new imaging technologies, ensuring that they meet safety and efficacy standards before they are widely adopted. This is particularly relevant in modalities such as digital radiography and CT, in which there are constant technological advances.

Although quantitative methods provide objective information, qualitative assessments remain an indispensable part of radiology practice^(6)^. Subjective assessments of image noise and artifacts by expert radiologists are crucial to determining the clinical adequacy of images. As shown in our results, techniques such as visual assessments and peer reviews complement objective metrics, ensuring that technical performance and clinical utility are both considered^(25)^.

The use of qualitative assessments in the identification of artifacts, for example, allows the detection of motion or metal artifacts, which can compromise diagnostic accuracy. In practice, qualitative opinions can guide modifications in imaging protocols, the calibration of systems, and the improvement of detectors^(12,25)^.

One of the main contributions of this study is to demonstrate the importance of synergy between quantitative and qualitative methodologies. Integrating qualitative expert assessment with robust quantitative analysis ensures a more comprehensive assessment of image quality. This balanced approach leads to improved imaging protocols, greater diagnostic accuracy, and increased patient safety.

## CONCLUSION

Metrics developed for image quality analysis are essential tools in radiology practice, especially with the advancement of diagnostic imaging methods. Understanding these tools allows radiologists to improve standards of patient care, as well as driving technological innovations in diagnostic radiology.

In this article, we have addressed a significant gap in the current literature by providing practical and accessible guidance on the main approaches to image quality assessment, emphasizing the importance of improving the diagnostic accuracy and safety of radiological procedures. We have also highlighted the need for ongoing research, continuing education, and technological developments to achieve these goals. This constitutes an invitation to the radiology community to actively seek a deeper understanding and practical applications of these methodologies, striving for excellence in patient care and the continuous advancement of the specialty.
